# Oxidative Stress Function in Women over 40 Years of Age, Considering Their Lifestyle

**DOI:** 10.3389/fendo.2017.00048

**Published:** 2017-03-16

**Authors:** Maria Paula Gonçalves Mota, Zirlene Santos, Jorge Soares, Ana Pereira, Sandra Fonseca, Francisco Peixoto, Isabel Gaivão, Maria Oliveira

**Affiliations:** ^1^Research Centre in Sports, Health and Human Development, Vila Real, Portugal; ^2^University of Trás-os-Montes e Alto Douro, Vila Real, Portugal; ^3^Faculty Metodista Granbery, Juiz de Fora, Brazil; ^4^Department of Sport Science, School of Education, Research Centre in Education, Polytechnic Institute of Setubal, Setubal, Portugal; ^5^Centre for Research and Technology of Agro-Environmental and Biological Sciences, Vila Real, Portugal; ^6^Animal and Veterinary Research Centre, Vila Real, Portugal; ^7^Chemistry Research Centre, Vila Real, Portugal

**Keywords:** aging, lipid peroxidation, DNA damage, oxidative stress, lifestyle, cardiovascular fitness

## Abstract

Aging is dependent on biological processes that determine the aging of the organism at the cellular level. The Oxidative Stress Theory of Aging might explain some of the age-related changes in cell macromolecules. Moreover, exposome and lifestyle may also induce changes in cell damage induced by oxidative stress. The aim of the present study was to analyze the related redox changes in lymphocyte function of healthy women over 40 years old. Three groups: younger (YG: 40–49 years), middle aged (MAG: 50–59 years), and older (OG: ≥60 years) were evaluated on anthropometric variables, blood pressure, cardiovascular fitness, lifestyle habits, perceived stress, DNA damage, malondialdehyde, catalase activity, and total antioxidant capacity. Physical activity and cardiovascular fitness were significantly higher in YG and MAG as compared to the OG. Systolic blood pressure increased significantly with group age. Frequency and total amount of alcohol intake were lower in the OG and higher in the MAG. No significant differences were observed between the three groups in oxidative stress parameters. Only alcohol consumption was associated with the higher DNA FPG-sensitive sites, and only in the YG (*p* < 0.05). Healthy lifestyle is critical to avoiding major ailments associated with aging. This may be inferred from the lack of significant differences in the various oxidative stress parameters measured in the healthy women over the age of 40 who took part in the study. Conscious lifestyle behaviors (decrease in alcohol and smoking habits) could have impaired the expected age-related oxidative stress increase.

## Introduction

Oxidative stress is involved in many degenerative processes (1), some of them related to the loss of function and aging (2). The aging theory of oxidative stress explains this process as a progressive accumulation of damaged macromolecules in cells with aging, due to an increased production of reactive oxygen species (ROS), which includes free radicals (atoms or molecules with an unpaired electron in their outer shell) that increase the risk of disease and death with advancing age. This increase in the oxidative stress on cells and tissues may be aggravated by a decrease in antioxidant capacity as well as reduced repair capacity (2, 3). Oxidative damage can result from the imbalance between prooxidants and antioxidants that decrease functional cellular processes with advancing age (4).

Several studies have also demonstrated the effects of environmental factors (e.g., radiation, pollution, etc.) and the role of daily life habits (smoking, diet, physical activity, daily stress, and alcohol) in oxidative stress (5, 6). For example, continuous exposure to exogenous pollutants or cigarette smoke causes lung injury by several mechanisms including the depletion of glutathione and other antioxidants, the initiation of redox cycling mechanisms, enhancement of the respiratory burst in neutrophils and macrophages, inactivation of protease inhibitors, and direct damage to lipids, nucleic acids, and proteins (7). It is also known that alcohol is involved in many processes causing oxidative stress, such as changes in the NAD^+^/NADH ratio in the cell as a result of alcohol metabolism, production of acetaldehyde, the interaction of which with proteins and lipids can result in radical increase and cell damage, and mitochondria damage and impaired energy production (8). Another possible lifestyle factor that may induce an increase in oxidative stress is daily stress. In fact, Schiavone et al. (9) suggest that the situation of daily stress can lead to an increase in brain oxidative stress and pathogenesis of neurologic and psychiatric diseases. On the other hand, regular physical activity may reduce oxidative stress and cell damage, as well as increase mitochondria efficiency, which results in lower ROS production, increase ATP production and antioxidant capacity (8). Diet, if balanced, can also reduce oxidative stress on cells and tissues, or increase if unbalanced. Literature is consistent either in enhancing the relationship between caloric restriction and reduced oxidative damage (10) or between increased caloric intake and increased oxidative stress and disease development process (11).

Given these studies, it seems reasonable to consider those variables related to lifestyle that could influence changes in oxidative stress parameters associated with aging. The Oxidative Stress Theory of Aging explains aging at the molecular level and alongside longevity or lifespan has been used for several years to study aging. Increased ROS levels can result in positive effects, including on the cellular processes that limit lifespan (5) because gradual increase in oxidative damage should intensify stimulation of stress-response that gradually increases the generation of ROS and age-dependent diseases. This type of analysis, hormesis mechanism, becomes even more important in older people, particularly in women, whose neuroendocrine age-related changes affect various organs, general function, and metabolic and antioxidant capacity (12). Considering this, the purpose of this study was to examine the related redox changes in function of exposome and health span in women over 40 years old. We hypothesized that variables such as smoking, alcohol consumption, daily caloric intake, perceived stress, weekly physical activity, cardiovascular fitness, and blood pressure could influence age-related changes in oxidative stress parameters.

## Subjects and Methods

### Subjects

Sixty-two healthy Caucasian women older than 40 years were divided into three groups: younger (YG: 40–49 years; *n* = 28), middle aged (MAG: 50–59 years; *n* = 21), and the older (OG: ≥60 years; *n* = 13). Lifestyle habits were evaluated in all candidates with an individual interview (face-to-face questionnaire) evaluated by a physician. Inclusion criteria include women (≥40 years) without health problems that could influence cardiorespiratory exercise test, and exclusion criteria comprised pacemakers, metallic prosthesis implants, walking only with assistance, and physiological conditions known to disturb the musculoskeletal system. All specific parameters were measured in previous studies by an experimental physiologist in order to ensure that all protocols could be repeated accurately. A written informed consent was obtained from each participant for the permission to use their information for the present report, and the experimental procedures were approved following the Helsinki Declaration and have been performed with the approval and Ethics Committee of Research Center in Sports, Health and Human Development, University of Trás-os-Montes and Alto Douro (number 052012). No vulnerable populations were involved.

### Methods

#### Assessment of Lifestyle Habits

For all participants who chose to enroll in this study, after obtaining their informed consent, their responses about life habits were maintained confidential and anonymous. Each subject was interviewed individually.

##### Food Frequency Questionnaire (FFQ)

The FFQ was filled out by the participants. The validation process for this questionnaire was described in detail elsewhere (13, 14). This questionnaire includes 131 food items with specified serving sizes described using standard volume and weight or natural portions (e.g., one orange, two slices of bread). Participants filled out the questionnaire items based on their average frequency of consumption over the past year. The selected frequency category for each food item was converted into daily caloric intake (kilocalories per day) and alcohol intake (grams per day).

##### Questionnaire of Physical Activity (IPAQ)

The participants completed the short version (15) of IPAQ, translated and validated for the Portuguese population (16). The questionnaire included nine IPAQ items, which ask for information about walking and moderate and vigorous physical activity in the last 7 days. Calculation of the total score requires summation of the duration (in minutes) and frequency (days) of walking, moderate-intensity and vigorous-intensity activity, and allows the classification of three levels of subjects’ total daily physical activity: insufficiency active (achieving less than 1,500 MET-min/week), minimally active (achieving a minimum of at least 1,500 MET-min/week), and active (achieving a minimum of at least 3,000 MET-min/week).

##### Perceived Stress Scale (PSS)

All participants completed the questionnaire, which is a 10-item form questionnaire, validated for the Portuguese population (17). The PSS-10 items refer to events that occurred during the last month. Participants responded on a 5-point scale ranging from 0 (*never*) to 4 (*very often*). Of the 10 items, 4 were worded in a positive direction, so they were reverse scored. The responses to the 10 items were then summed to create a psychological stress score, ranging from 0–40, with higher scores indicating greater psychological stress.

###### *Anthropometric* *Measure*

Height (cm) was evaluated using a stadiometer (Cabral, model 14), and body weight (kg) was measured using a digital scale (Philips, type HF 351/00). Subjects were measured while wearing shorts and t-shirts (shoes and socks were removed). Waist circumference was used with the umbilicus point used for reference (18).

###### *Cardiovascular* *Fitness (6-Minute Walking Test)*

It is a practical simple test that measures the distance that a person can quickly walk on a flat, hard surface in a period of 6 min. This test evaluates the global and integrated responses of all systems involved during exercise, including the cardiorespiratory, neuromuscular, and muscle metabolism (19) and assesses the submaximal level of functional capacity. Patients choose their own maximal intensity of exercise and are allowed to stop the test. Thus, most of the patients do not achieve maximal exercise capacity. Considering that most daily living physical activities are performed at submaximal intensity levels, this test may better express the functional exercise level for daily physical activities.

###### *Blood* *Pressure*

Participants were advised to remain seated for about 10 min, then systolic and diastolic blood pressure were measured using the OMRON M2 (Hem: 7117—E) device.

#### Oxidative Stress Parameters

The participants were instructed not to perform exercise on the day before blood collection and to arrive in the morning in fasting condition. 10 mL of blood were collected by venous puncture into the vacuum K_3_EDTA tubes. Blood sample of each subject was added to phosphate buffer solution (PBS) (1v:1v), gently mixed, and carefully slid into a tube containing Histopaque 1077 (Sigma) (2v:1v). The sample was then centrifuged 700 × *g* for 20 min at 4°C. Lymphocytes were retrieved from just above the boundary between PBS and Histopaque using a pipette and washed in PBS twice. The supernatant was then removed as much as possible using a pipette, with cells on the pellet re-suspended in 280 μl of 1% low melting point agarose (Gibco) in PBS at 37°C to perform Comet assay. The plasma was separated and stored in Eppendorf tubes at −80°C for future analysis.

##### Total Protein Determination

Total protein concentration was measured in plasma according to biuret method by spectrophotometry, using serum albumin as the standard (20).

##### Malondialdehyde (MDA)

The levels of lipid peroxides were estimated based on the thiobarbituric acid reactive substances (TBARS) method, with some modifications (21). Plasma samples of 100 μL were taken and mixed with 200 μL tricloroacetic acid (10%) and centrifugated at 16.000 × *g* (top speed) for 1 min. 200 μL of supernatant were taken and mixed with 200 μL of thiobarbituric acid (TBA) reagent (1% TBA). The mixture was heated at 80–90°C during 10 min and cooled down to room temperature for 20 min. Lipid peroxidation was estimated by the appearance of TBARS spectrophotometrically quantified at 535 nm. The amount of TBARS formed was calculated using molar extinction coefficient (ε) of 1.56 × 10^5^ M^−1^ cm^−1^, and the results were expressed as TBARS concentration (nanomoles per milligram of protein).

##### Total Antioxidant Capacity (TAC)

The TAC in plasma was determined using the ABTS radical-scavenging activity measured by a procedure previously reported (22), with slight modifications. A solution was prepared with ABTS^•+^ (7 mM) and potassium persulfate (140 mM) in 5 mL of distilled water. The solution was held at room temperature in the dark for 12–16 h before use. The ABTS^•+^ solution was diluted in acetate buffer (100 mM, pH 4.5) in order to obtain an absorbance of 0.7 at 734 nm. Fresh ABTS^•+^ solution was prepared for each analysis. To obtain Trolox equivalent, a standard solution was prepared at 0 (control), 1.25, 2.50, 5.00, 7.50, 10.00, 15.00, and 20.00 μM. To measure the antioxidant capacity of the samples, three different sample volumes were used. Sample antioxidant capacity was expressed in terms of Trolox equivalent activity.

##### Catalase Activity

The activity of catalase (CAT) in plasma samples was measured polarographically by the oxygraphic method using a Clark oxygen electrode (Hansatech, Norfolk, UK), according to Ref. (23). Changes in oxygen concentration were measured in the incubation medium made of 50 mM phosphate buffer, pH 7.4 and 10 mM H_2_O_2_. One unit of catalase produces 1 μmol O_2_ min^−1^.

##### DNA Damage

Comet assay, or single cell gel electrophoresis, was used to measure DNA strand breaks (DNA SBs). These protocol procedures followed the experiments performed by our research team, described elsewhere (24) according to Collins AR (25).

#### Statistical Analysis

Descriptive statistics (mean ± SD) were used. The data were subjected to exploratory data analysis, using the graphical methods of Box-and-Whiskers and Stem-and-Leaf plots, to identify and purge outliers that could significantly alter central trend parameters. The evaluation of symmetry and flatness of the distribution curves was made using the values of skewness and kurtosis, respectively. The normality of distributions was confirmed by the Kolmogorov–Smirnov test with Lilliefors correction.

Repeated-measures ANOVA was used to assess the differences between the groups. The Tukey test for significant differences was used for *post hoc* analysis. The influence of lifestyle habits in relation to age and oxidative stress parameters was analyzed in each group, comparing smokers with non-smokers, and alcohol consumers with non-consumers, by a *t*-test for independent samples. Statistical significance was set at *p* < 0.05 for all analyses. All data were analyzed using SPSS 17.0.

## Results

**Figure 1:**
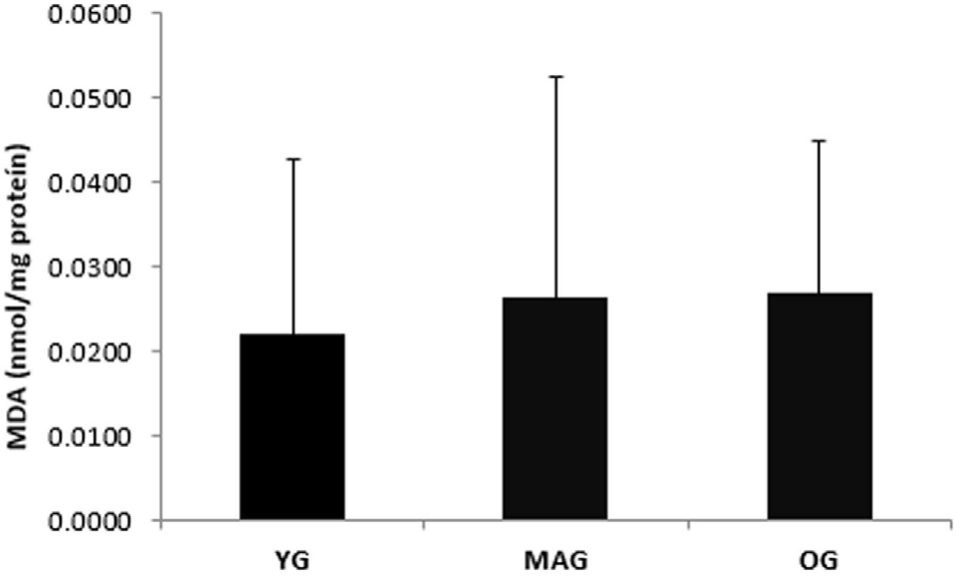
**Biomarkers of oxidative stress (MDA) (nanomoles per milligram protein) in the three groups (YG, younger; MAG, aged; OG, older)**. Mean values are shown with SD.

**Figure 2:**
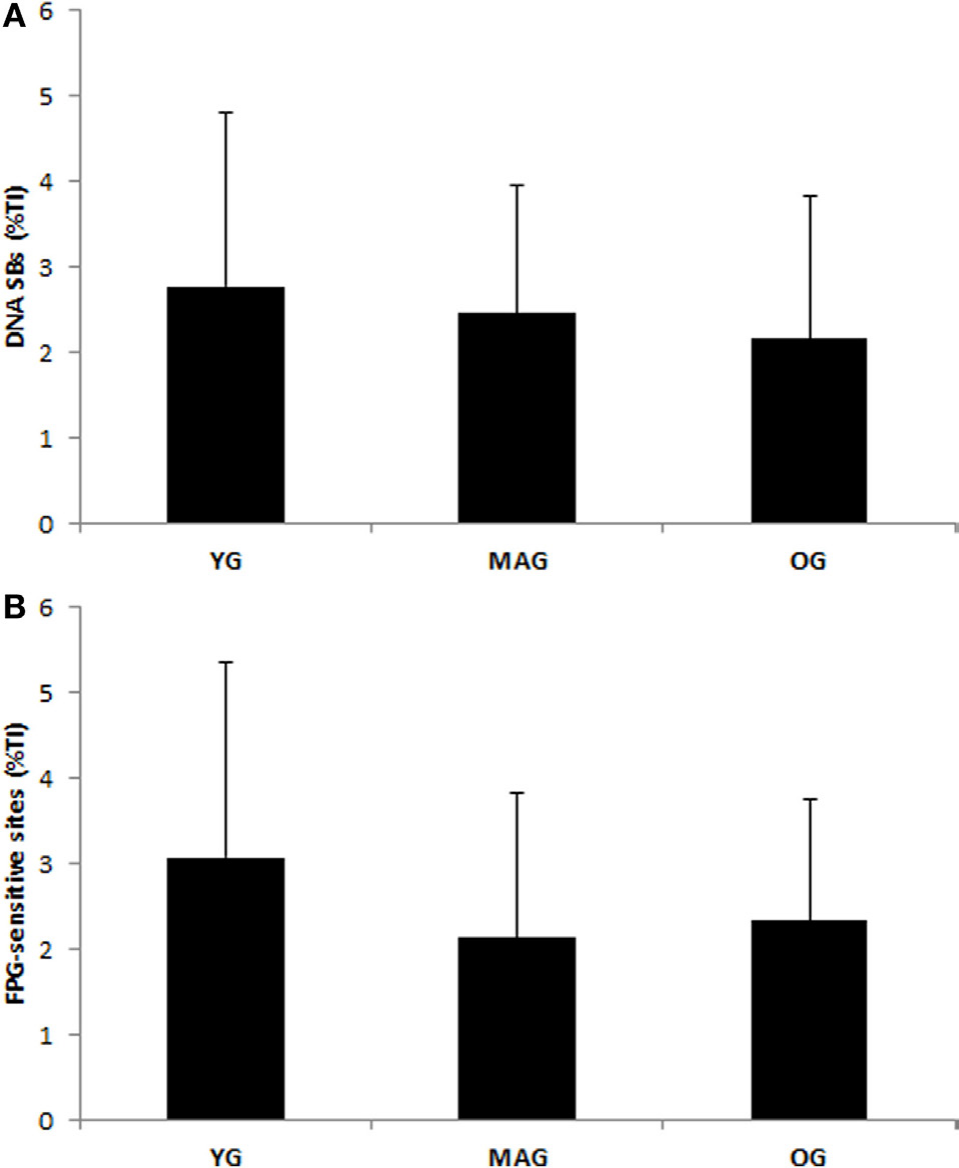
**DNA damage and DNA repair activity in lymphocytes measured in the three groups (YG, younger; MAG, aged; OG, older)**. **(A)** DNA strand breaks. **(B)** FPG-sensitive sites. Mean values are shown with SD.

**Figure 3:**
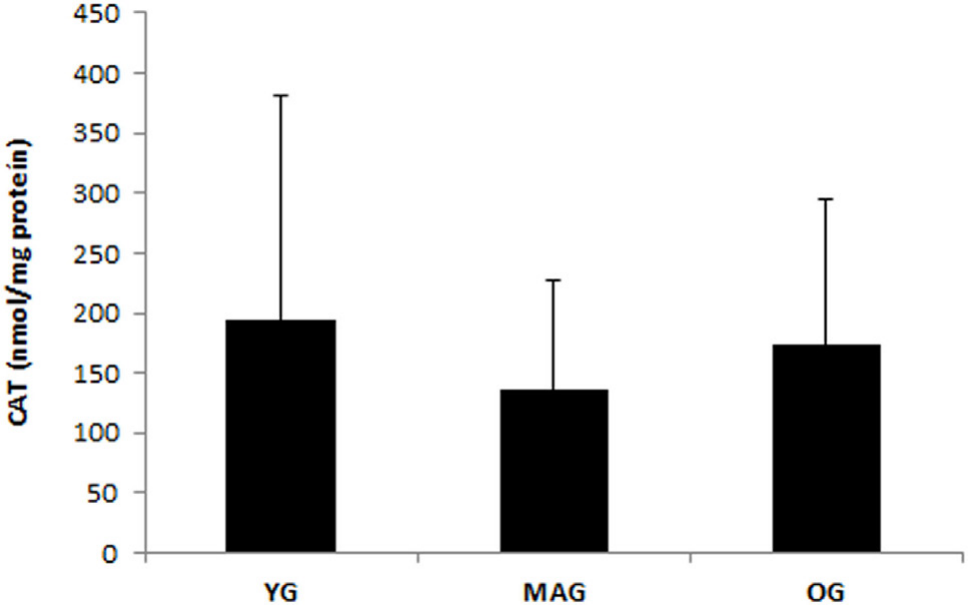
**Catalase activity in plasma for the three groups (YG, younger; MAG, aged; OG, older)**. Mean values are shown with SD.

**Figure 4:**
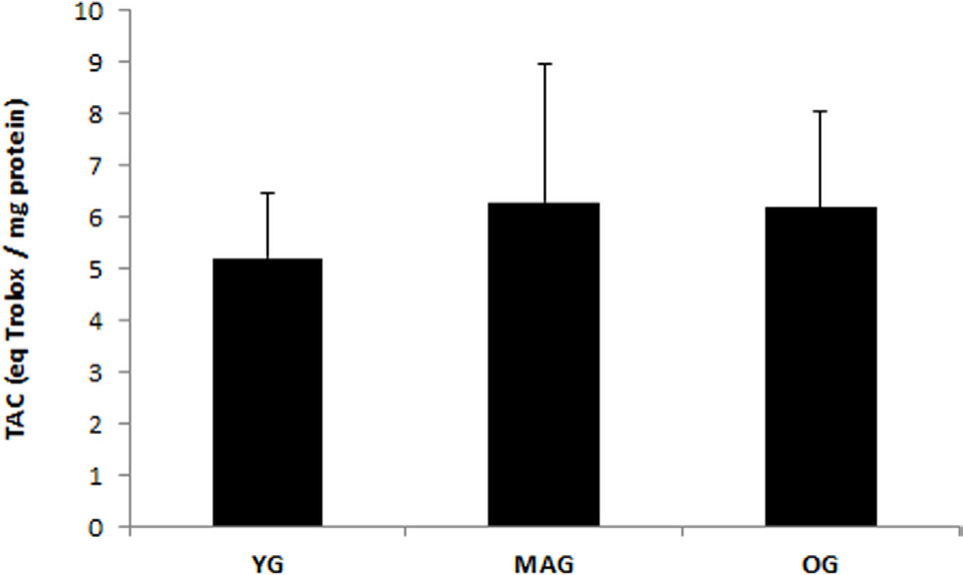
**Total antioxidant capacity (TAC) of the three groups (YG, younger; MAG, aged; OG, older)**. Mean values are shown with SD.

Considering group characteristics, only differences in age (*F* = 141.273, *p* = 0.000), height (*F* = 3.589, *p* = 0.035), weekly physical activity (*F* = 3.290, *p* = 0.045), and cardiovascular fitness (*F* = 6.410; *p* = 0.000) were found (Table 1). Regarding biochemical parameters, no significant differences in the plasma concentration of MDA (nanomoles per milligram protein) were observed (Figure 1). Nevertheless, it was possible to notice that achieved values tended to increase with age. No significant differences in DNA SBs damage of lymphocytes were found (Figure 2A), although the results showed a trend of decreasing with age. The differences in the concentration of DNA FPG-sensitive sites were not significant (Figure 2B). Their concentrations do not follow a linearity of values in relation to age group, having their highest values in the YG, followed by the OG. Although catalase activity in plasma presented its highest values in MAG and lowest in the YG, these differences were not significant (Figure 3). The concentration of ABTS showed no significant differences between groups; however, their highest values were observed in the MAG and the smallest in the YG (Figure 4). The error bars appear to have a large variation due to the individual differences between subjects. Comparing MAG and OG together with YG no statistically differences were found (*p* ≥ 0.05).

**Table 1:** **Mean values (±SD) of age, physical characteristics [weight, height, BMI, waist circumference (WC)], and functional capacity [6-minute walking test (6MWT), systolic blood pressure, and diastolic blood pressure] and daily life habits (caloric intake, weekly physical activity, alcohol intake, smoking habits, and perceived stress) of the three aged groups (YG, younger group, MAG, middle-aged group; OG, older group)**.

	YG	MAG	OG
	
Variables	Mean ± DP	Mean ± DP	Mean ± DP
Age (years)*	45.54 ± 2.41	53.95 ± 3.27	66.62 ± 6.36
Weight (kg)	66.74 ± 11.23	67.39 ± 10.17	69.56 ± 11.96
Height (cm)*	158.12 ± 5.36	156.31 ± 6.41	153.00 ± 5.32
BMI (kg/m^2^)	27.18 ± 4.29	27.55 ± 3.61	29.82 ± 5.74
WC (cm)	90.67 ± 1.11	89.94 ± 8.77	95.23 ± 11.01
6MWT (m)	609.93 ± 61.34	601.56 ± 40.78	538.54 ± 77.73
SBP (mmHg)*	119.85 ± 11.43	122.33 ± 8.88	138.69 ± 16.26
DBP (mmHg)	75.42 ± 6.84	75.38 ± 8.08	77.38 ± 8.57
Caloric intake (kcal/day)	2,006.97 ± 359.28	2,012.23 ± 442.47	1,993.05 ± 357.65
Physical activity (MET-min/week)*	916.17 ± 621.30	977.87 ± 651.85	461.77 ± 268.42
Alcohol intake (g/d) (% of drinking)^a^	5.81 ± 5.14 (60.0%)	6.63 ± 8.3 (70.6%)	3.96 ± 4.6 (55.6%)
smoking (cigarettes/d) (% of smokers)^a^	9.3 ± 3.4 (21.4%)	13.0 ± 7.1 (10.0%)	0
Perceived Stress Scale	16.5 ± 5.8	19.5 ± 7.5	17.1 ± 5.4

*^a^Only the average number of cigarettes per day between smokers and average alcohol consumption between drinkers*.

*Significant differences between groups (*p* < 0.05). Data presented are mean ± SD.

## Discussion

The present study examines the variance of oxidative stress in women over 40 years of age, with consideration to some of their lifestyle habits. Our primary results pointed out a lack of significant differences in the various oxidative stress parameters measured in the healthy women over the age of 40 who took part in the study. Contrary to expectations, and to the oxidative stress theory of aging (1), our results showed no significant changes with age in parameters of oxidative stress related to MDA, DNA SBs, FPG-sensitive sites, catalase activity, or TAC. Whereas the extent of variation in age was 42 years (from 40 to 82 years), spanning important hormonal changes associated with decreased antioxidant (26) protection, and progressive loss of function, we would expect a significant increase in oxidative stress at least in the OG. Indeed, Junqueira et al. (26) found a significant increase in MDA plasma concentration with age, from 20 to 45% in subjects aged over 50 years compared to the 20–29 age group. This increase in oxidative stress with aging was also documented by Ref. (27, 28), while others have provided controversial results (29, 30) and somewhat similar data to ours. Moreover, in a cross-sectional study (30), individuals of different age groups (25–29, 30–39, 40–49, 50–59, 60–69, and ≥70 years) revealed that oxidative stress markers were not related to age in individuals under 60 years. Furthermore, TAC only presented a related reduction in groups over 60 years, suggesting the increase in oxidative stress with age may become pronounced after 60 years.

Several factors related to lifestyle may have contributed to the lack of statistically significant differences on any of the oxidative parameters in our study. In terms of diet, Sanz and Stefanatos (31) reported that caloric restriction for a period of one year decreased the production of ROS. Our data evidenced a small trend to decrease caloric intake, from the YG to the OG. That should not, *per se*, explain the absence of the expected increase in oxidative stress parameters. However, in addition to metabolic imbalances related to intake/energy expenditure, it is necessary to take into account the composition of the diet (not considered in this study) and other risk behaviors such as smoking and alcohol consumption. The latter two are highly related to increased oxidative stress (32). Moreover, although there were no evidenced significant differences between the three groups, one can also perceive a trend to reduce smoking habits and alcohol consumption, from the YG to the OG, suggesting a greater awareness and adoption of a healthy lifestyle over time. So, although quantitatively the reduction in the number of cigarettes smoked per day may have not been significant, it could have been enough to prevent antioxidant depletion, redox cycling mechanisms, initiation of inflammatory response, and other ROS damage induced to lipids, nucleic acids, and proteins (7). Moreover, a study comparing smokers and non-smokers (33) showed that superoxide dismutase, glutathione peroxidase, and catalase were significantly higher in non-smokers compared to smokers. In the present study, the reduction in smoking may have thwarted the OG’s tendency to reduce or increase antioxidant status and oxidative damage.

In relation to the high consumption of alcohol (34), claim that ethanol metabolism is involved in the depletion of the antioxidant system components and is directly linked to the generation of ROS and oxidative stress in the liver and decreases antioxidant capacity. In our study, the older group evidenced a lower alcohol intake than the others. Considering that this group was the one that usually evidences higher concentrations of oxidative damaged macromolecules (31), the combined effects of smoking habits and alcohol intake reduction may have influenced the lack of alteration of oxidative stress damage with age (35, 36). Although some changes in daily physical activity with age were observed that could contribute to an increase in oxidative stress, our sample revealed both a low daily activity and low stress perception in all groups.

Further studies including subjects over 80 years and considering menopause status are recommended. Nevertheless, the present study has some limitations, specifically in the group over 60 years, which is very small for this kind of study. Also, we did not analyze one specific young group (between 20 and 30 years), and it is possible that an early inappropriate lifestyle can affect oxidative stress function before 40 years of age.

In summary, the present study showed no significant changes in parameters of oxidative stress with age in healthy women aged over 40 years. A trend was observed to reduce smoking habits, alcohol consumption, and caloric intake with age, which may explain the absence of significant changes in parameters of oxidative stress. These results may be important to emphasize the importance of healthy behaviors in order to decrease the age-related dysfunction promoted by ROS.

## Author Contributions

MM: data collection and scientific supervision. MM, ZS, and JS: study design. FP, IG, and MO: biochemical analysis. JS and AP: supervision and collection of physical tests battery. AP and SF: data collection and monitoring of variables of social sciences. ZS and JS: organization of the database and statistical treatment.

## Conflict of Interest Statement

The authors declare that the research was conducted in the absence of any commercial or financial relationships that could be construed as a potential conflict of interest.
